# Gut Microbiota, Inflammation, and Probiotic Supplementation in Fetal Growth Restriction—A Comprehensive Review of Human and Animal Studies

**DOI:** 10.3390/life13122239

**Published:** 2023-11-21

**Authors:** Naser A. Alsharairi, Li Li

**Affiliations:** 1 Heart, Mind and Body Research Group, Griffith University, Gold Coast, QLD 4222, Australia; 2 School of Science, Western Sydney University, Richmond, NSW 2753, Australia; l.li7@westernsydney.edu.au

**Keywords:** fetal growth restriction, gut microbiota, inflammation, probiotics, pregnancy, fetus

## Abstract

Fetal growth restriction (FGR) is a pathological state that represents a fetus’s inability to achieve adequate growth during pregnancy. Several maternal, placental, and fetal factors are likely associated with FGR etiology. FGR is linked to severe fetal and neonatal complications, as well as adverse health consequences in adulthood. Numerous randomized controlled trials (RCTs) have demonstrated improved growth in FGR fetuses with promising treatment strategies such as maternal micronutrient, amino acid, and nitric oxide supplementation. Elevated inflammation in pregnant women diagnosed with FGR has been associated with an imbalance between pro- and anti-inflammatory cytokines. Gut microbiota dysbiosis may result in increased FGR-related inflammation. Probiotic treatment may relieve FGR-induced inflammation and improve fetal growth. The aim of this review is to provide an overview of the gut microbiota and inflammatory profiles associated with FGR and explore the potential of probiotics in treating FGR.

## 1. Introduction

Fetal growth restriction (FGR), also termed intrauterine growth restriction (IUGR), is a state that reflects the failure of a fetus to attain its full genetic growth at a particular gestational age. This means that the baby weighs less than 9 out of 10 babies of the same gestational age [1,2]. FGR is diagnosed using ultrasound and described by an estimated fetal birth weight (EFW) or abdominal circumference (AC) below the threshold of the 10th percentile for gestational age [1,2]. FGR is often used interchangeably with small for gestational age (SGA) (determined as a weight/length of less than two standard deviations under the mean for gestational age) but they are not the same, as SGA has the clinical features of malnutrition and in utero growth retardation [1,3,4]. SGA infants may have FGR, but SGA cannot be used as a proxy for FGR, and not all SGA infants are pathologically growth-restricted [5].

FGR is classified into two categories: symmetric and asymmetric. Symmetric FGR (hypoplastic small for date) is identified in early pregnancy in about 20% to 30% of FGR cases, and is characterized by a reduction in the size of all organs, resulting from fetal nutrient restrictions, poor placental function, and autosomal chromosome aberrations (e.g., aneuploidy). TORCH infections (including toxoplasmosis, rubella, cytomegalovirus, and herpes) are associated with symmetrical FGR. Asymmetric FGR (malnourished babies) manifests in the late second/third trimester of pregnancy in a large fraction of FGR cases (70–80%) and is caused by preeclampsia (PE) due to utero-placental insufficiency. Asymmetric FGR is characterized by a reduced abdominal circumference (fetal liver), while the head circumference (fetal brain) is of average size. The decrease in liver size results in a depletion of adipose tissue and reduced glycogen storage, as well as total blood flow to the fetus [2,3,4,6]. Infants with symmetric FGR have poor prognosis compared to those with asymmetric FGR. In symmetric FGR, the body’s cell numbers are usually abnormal and reduced in early pregnancy, which leads to fetal growth abnormalities [2,3,6].

The etiology of FGR is related to a myriad of factors. The maternal factors include low socioeconomic status, smoking, alcohol, antineoplastic agents, PE, anemia, hypertensive disorders, systemic lupus erythematosus, chronic renal disease, antiphospholipid syndrome, respiratory diseases, cystic fibrosis, gastrointestinal diseases, and infections [1,2,3,4,6,7,8,9,10]. There are several factors related to the placenta. These include low placental weight, small terminal villi, infections, and confined placental mosaicism. Fetal anomalies, including chromosomal abnormalities, genetic syndromes, congenital anomalies/infections, and metabolic disorders, also play a role in FGR [1,2,3,4,6,7,8,9,10]. Epigenetics may also contribute to FGR, which involve changes in gene expression resulting from DNA methylation, non-coding RNAs, and post-translational modification of histones. Environmental factors (e.g., smoking, alcohol) have been found to shape these epigenetic changes, in which many FGR-related gene expressions are involved in fetal phenotype and placental epigenome alterations [11].

FGR increases insulin sensitivity and decreases counter-regulatory hormones, intestinal perfusion, subcutaneous fat stores, and the body’s protein/nitrogen contents. Infants with FGR have shown a significant reduction in the uptake of nutrients (e.g., minerals, amino acids, glucose), likely as a result of placental insufficiency, which, in turn, can lead to chronic hypoxia and reduce glycogen stores in the muscles and liver [2,3]. FGR infants are at a significant risk of developing health consequences later in life. These include cardiovascular (e.g., coronary disease, heart failure, early-onset atherosclerosis), metabolic (e.g., fatty liver disease, type 2 diabetes, dyslipidemia), and respiratory consequences (e.g., asthma, impaired lung function, restrictive pulmonary disease) [3,12,13]. Oxidative stress (OS) and dysregulated genes related to inflammation have been postulated to explain the increased risk of developing these diseases [12,14]. FGR and its associated diseases are influenced by multiple molecular mechanisms, including transforming growth factor β (TGF-β), protein 53 (p53) phosphorylation, the mechanistic target of rapamycin (mTOR), nucleotide oligomerization domain receptor 1 (NOD1), heat-shock proteins, glucocorticoids, and leptin [14].

The early detection of pregnant women at high risk of developing FGR would allow the application of promising treatment strategies to improve fetal growth. Several randomized controlled trials (RCTs) have shown that FGR-specific treatments, such as maternal micronutrient and amino acid supplementation (iron, zinc, calcium, magnesium, N-acetyl cysteine/L-Arginine), maternal nitric oxide supplementation, maternal growth hormone supplementation, aspirin, antiplatelet/anticoagulant agents, calcium channel blockers, proton pump inhibitors, and melatonin/heparin, can improve birth weight in FGR pregnancies [15,16,17,18,19,20,21,22,23,24]. These treatments appear to largely reflect the current understanding of the etiology of this condition.

Complicated pregnancy is a state where aberrant inflammation can occur due to an imbalance between pro- and anti-inflammatory cytokines/chemokines [25,26,27]. Aberrant inflammation can lead to an increased risk of FGR and other adverse pregnancy outcomes such as obesity, inflammatory bowel disease (IBD), gestational diabetes (GDM), PE, pregnancy loss, and autoimmune diseases [27]. Pregnancy is a period where the infant gut microbial composition undergoes changes influenced by several factors, including gestational age, lactation stage, maternal diet, antibiotic exposure, and mode of feeding/delivery [28,29]. Gut microbiota dysbiosis during pregnancy is a potential contributing factor to metabolic inflammation and has been associated with many diseases in pregnant women and infants, including endometriosis, environmental enteric dysfunction, PE, GDM, intrahepatic cholestasis, hyperemesis gravidarum, necrotizing enterocolitis (NEC), neonatal diabetes (NDM), obesity, and asthma [30,31,32,33,34,35,36,37]. Gut-derived microbial components such as lipopolysaccharides (LPS) have been proposed as an underlying mechanism for this effect [38,39].

Probiotic supplementation has been shown to modulate aberrant gut inflammation in the early years of life, thereby providing them with a potential role in the treatment of inflammation-related diseases [30,31,32,33,36]. Probiotic strains, such as short-chain fatty acids (SCFAs) producing *Bifidobacterium* and *Lactobacillus*, may have the potential to improve immune responses and alleviate inflammation by reducing the production of pro-inflammatory cytokines in infant intestinal epithelial cells [30,31,32,33]. A recent review of RCTs suggests that probiotic supplementation may offer beneficial effects in reducing the risk of PE in pregnant women [40]. However, no comprehensive reviews on gut microbiota community profiles, inflammatory markers, and the potential impact of probiotic use in FGR are available. Thus, this review aims to summarize the existing human and animal studies on gut microbiota and inflammatory profiles linked to FGR and the effect of probiotics as a potential therapeutic approach.

## 2. Methods

The PubMed/Medline database was searched to identify the relevant studies that were published over the last 20 years focusing on intestinal microbiota composition, inflammatory profiles, and probiotic supplementation in FGR. The literature search was conducted using the following keywords: FGR, IUGR, gut microbiota, inflammation, inflammatory markers, and probiotic supplementation. No restrictions on the study design were applied to the search. 

## 3. Gut Microbiota in FGR

Only a few animal [41,42,43,44] and human [45,46,47,48,49,50] observational studies were found to report the role of gut microbiota in FGR. The gut microbiomes in neonate animal models were [41,42,43,44] reported more often than the maternal gut microbiomes [42]. The human studies mainly investigated the maternal gut microbiomes [46,47,48], with one study comparing those between infant twins grouped based on chorionicity and discordance in birth weight [45]. Despite the difference in focus, together, these studies depict the mediating role that gut microbiota play in either manifestations of FGR in infant growth and health or the development of FGR in the gravidas. The taxonomic and structural changes in the gut microbiota are reported in all studies found. The functional capacity of the gut microbiomes [43,44,45,46] and gut microbial metabolic changes [41,42,45] were also explored.

### 3.1. Animal Models

The impacts of FGR on various genera of the class *Clostridia* and potentially beneficial and pathogenic genera in rodent pups were revealed in one early [42] and one more recent [41] study. The early study reported that the caecocolonic densities of the SCFAs producers clostridial cluster IV (including the *Clostridium leptum* “*C. leptum*” cluster and *Faecalibacterium prausnitzii* “*F. prausnitzii*”), and the *Roseburia intestinalis* “*R. intestinalis*” cluster, were significantly more abundant in FGR pups than controls matched by age and feeding conditions at 5 days of age [42]. The extent of the increase in densities of total bacteria and the aforementioned taxa, together with SCFAs producing clostridial cluster XIVa, however, appeared to be smaller in FGR pups during days 5–12. Their densities became significantly lower in FGR pups than controls at day 12, as did the relative abundance of clostridial cluster XIVa. This broadly aligns with the observation by Arai et al. [41], who reported significantly lower relative abundances of two clostridial cluster IV genera *Clostridium* and *Eubacterium*, unclassified *Clostridiales* members, and *Allobacutum*, a butyrate producing genus [51], when compared with controls matched by age and feeding condition at the age of 2 weeks. Rat pups of the same breed in both studies were weaned off on day 21. Isocaloric diets with 8% and 7% protein content were used by the early [42] and more recent [41] study, respectively, to induce FGR confirmed by the same weight-based approach. Despite this broad consistency, an SCFA-producing clostridial cluster of the XIVa genus *Blautia* was elevated in FGR pups in the more recent study [41]. This slight inconsistency between the two studies was partly reflected in the changes in SCFAs levels. The early study found significantly lower levels of total SCFAs, acetate, propionate, minor SCFAs, and an insignificant reduction in the levels of butyrate in the caecocolonic content of FGR pups when compared with the controls at day 12, followed by significantly lower gene expression related to butyrate uptake one day after weaning in FGR pups than controls [42]. The more recent study reported significantly lower levels of acetate, and an insignificant reduction in propionate with no difference in minor SCFAs between caecal content of FGR and control groups at the age 2 weeks of age [41]. A significantly higher level of butyrate was, however, observed in the FGR group. Interestingly, the early study highlighted the higher butyrate producing capacity of the caecocolonic content of the FGR group sampled at day 12 in a further in vitro fermentation experiment [42]. In vivo findings showed a significant decrease in body weight in the rat guts colonized with Ruminococcus, *Roseburia*, *Butyricicoccus*, and *Bacteroidota* at the age of 6 and 9 weeks [52]. It is unclear whether the presence and absence of the butyrate producer *Allobacutum* in Arai et al. [41] and Fança-Berthon et al. [42], respectively, could partly account for the differences in SCFA levels between the two studies. The two studies also differed in their bacterial quantification methods and sample sizes. Despite inconsistencies in the differences in quantities or relative abundances of potentially beneficial and pathogenic taxa between the two studies, both identified significantly suppressed levels of potentially beneficial taxa, such as *Bifidobacterium,* both before and well after weaning [42], and the Verrucomicrobia phylum that contains the potentially beneficial genus *Akkermansia* before weaning [41], in FGR pups when compared with the controls. The more recent study also reported significantly higher relative abundances of potentially pathogenic *Enterococcus* and the phylum Proteobacteria that contains many potential pathogens in the second week of life in the FGR group [41].

In vivo findings showed that low-birth-weight piglets harbored lower relative abundances of SCFA producing bacteria, such as Prevotellaceae, Ruminococcaceae, *Alistipes*, Bacteroidetes, and *Blautia*, when compared with normal birth weight controls, during the first 35 days of life. The colonic contents of acetate, propionate, and butyrate produced by these bacteria were all decreased [53]. The elevation of potentially pathogenic taxa was observed in a study investigating small intestinal microbiomes in FGR piglets in comparison with controls matched by age and feeding condition [43]. Significantly higher abundances of Proteobacteria and *Pasteurella* were found in the ileal content at weaning, and *Escherichia-Shigella* in the jejunal content 7 days after weaning. The opposite was observed for relative abundances of Bacteroidetes, *Bacteroides*, the clostridia cluster IV genus *Oscillibacter* in jejunum, and *Firmicutes* in ileum at weaning. Those of Bacteroidetes and *Bacteroides* in jejunum were also suppressed well before and after weaning. Contrary to this, an enrichment of *Bacteroides* was reported in FGR rodent pups well before weaning [42]. In addition to compositional differences between FGR piglets and the corresponding controls, alpha diversity in the jejunum appeared to be significantly lower as represented by the Chao1 index well before weaning and by the Shannon index at weaning [43]. The reduced bacterial density and diversity, and the compositional alteration demonstrated in the FGR animal models when compared with the controls, likely indicate disrupted gut colonisation after birth [42], and subsequent dysbiosis after stabilization of the microbiome [43]. The etiology underlying this alteration has been recognized. Crucial anatomical and functional disruption to the intestine have been highlighted in animal models [42,54]. Examples of anatomical changes in FGR include decreased intestinal length and weight, impaired intestinal mucosa development, and proliferation capacity. These appear to correspond with several intestinal functional changes. Examples include suppressed intestinal absorption capacity of various compounds such as butyrate, and impaired or delayed digestive enzymes such as jejunal lactase [42] and maltase [54] activity.

The immediate and long-term sequelae of the disrupted gut colonization and dysbiosis seem to be manifested in host energy metabolism and host physiological growth. The microbiome-associated functional potential of macronutrient metabolism, glycan synthesis and metabolism, and xenobiotics biodegradation and metabolism appear to be suppressed [43]; together with an altered gut microbiome structure and intestinal anatomy and function [41,42]. The availability of SCFAs has been considered as a critical factor impacting host growth and health [41,42,43,44]. For example, body weight was found to positively correlated with the relative abundances of taxa known for SCFAs production from one or more macronutrients, including *Bacteriodes*, *Oscillibacter*, and Ruminococcaceae-UCG-002 in the small intestinal content of FGR piglets before the age of 28 days [43]. The same study also reported negative correlations between body weight and the relative abundances of potentially pathogenic and pro-inflammatory taxa, including Proteobacteria and *Escherichia-Shigella* in the same time frame. In vivo experiments have revealed that low-birth-weight pigs had a much lower growth rate than normal-birth-weight pigs. Pigs classified as having a “poor” average daily weight gain had significantly lower abundances of Ruminococcaceae UCG-005, Prevotellaceae, and *Lactobacillus* at the age of 4–23 days [55]. Importantly, a study by Xiong et al. [44] on the same breed of swine indicated it was possible to reverse alterations in alpha diversity and several bacterial taxa in the small intestinal microbiome of FGR pigs much later after weaning when nutrition was adequate. For example, FGR pigs appeared to present a significantly higher alpha diversity in jejunal and ileal content when they grew to 50 kg and 100 kg (approximately aged 4 months and 6 months). A significant reduction in Proteobacteria and the augmentation in Firmicutes in jejunum were found in pigs grown to 50 kg and 100 kg. The latter may have corresponded to an enrichment of microbial functional capacity in carbohydrate metabolism and glycan synthesis and metabolism, supporting host intestinal utilization of SCFAs for energy harvest and other health benefits. However, this microbiome-associated compensation later in life appeared to be insufficient to entirely reverse the restriction in growth and development. Other findings from this study seemed to support the earlier FGR piglet study. The clear differences in beta diversity between FGR pigs and controls supported the observation that the overall structure of the small intestinal microbiome was altered in FGR swine and remained so much later after weaning. The positive correlation between several of the growth indicators and the relative abundance of unclassified Ruminococcaceae broadly aligned with a similar observation for the SCFA producer Ruminococcaceae by Zhang et al. [43], thus implying their role in compensating for FGR after birth [44]. The microbial functional capacity in amino acid and lipid metabolism remained suppressed much later after weaning. This highlights the need to evaluate the efficacy of corresponding dietary supplementation to help improve growth and health outcomes related to FGR. The potential role of FGR in regulating intestinal microbial gene expression is also worthy of investigation. This implies the potential influence of FGR on a wider range of metabolic pathways that may eventually modify host health outcomes. It should be noted that changes in the structural and functional potential of the gut microbiome in FGR piglets during growth and development after birth seemed to be accompanied by changes in host plasma hormones levels that regulate metabolism and growth until approximately 6 months after birth. The interrelationship between gut microbiota and host hormone levels, and the influence of such relationships on FGR manifestation, await clarification. Table 1 lists findings from animal models that have evaluated the alterations in gut microbiota in FGR cases.

### 3.2. Human Studies

Findings from one human study investigating gut microbiome in neonates with FGR partly shared a couple of similarities with those from the animal models [45]. The alpha diversity of gut microbiome immediately and a few days after birth was statistically indifferent among twins irrespective of chronicity and concordance in birth weight. However, a significantly higher alpha diversity of the gut microbiome was demonstrated in weight-discordant twin pairs with more severe forms of FGR in comparison with weight-discordant twin pairs with less severe forms of FGR and those with normal weight. This difference narrowed down and disappeared within a few days after birth. It is thus implied that the extent of exposure to adverse intrauterine environment, as in the case of FGR, is associated with influences on gut bacterial colonization, but this alteration appears to be restored within the early days of life. This ‘restoration’ in gut microbiome seems to align with that observed in the swine model from Xiong et al. well after weaning [44]. Among the genera that were negatively correlated with the severe consequences of FGR, increased body weight and average daily gain, the diminished levels of *Enterococcus* and two genera from Proteobacteria (*Acinetobacter* and *Actinobacillus*) contradicted the findings in the rodent and swine models [41,43]. The differences in *Enterococcus* and *Acinetobacter* associated with severity of FGR also appeared to disappear within the first few days of life [45]. This restoration was likely beneficial as the suppressed levels of *Enterococcus* and its representative species *E. faecium* are associated with the depletion of fecal methionine and cysteine, which participate in anti-oxidative processes. This could alleviate OS, leading to common abnormalities in the maternal–placenta–fetal circulation, and thus impairing neuro-behavioral development. The enrichment of butyrate producers *Oscillospira* and *Coprococcus* in those with FGR coincided with the higher butyrate levels found in FGR rodent pups by Arai et al. [41], and thus possibly implies another compensation mechanism, which may be important in partially restoring energy metabolism and regulating inflammatory processes in neonates with FGR [45]. An RCT has shown that a resistant starch enriched diet (maize) during pregnancy increases the relative abundances of butyrate-producing starch-resistant degrading bacteria (*Ruminococcus bromii* and *F. prausnitzii*) involved in energy metabolism, signaling, and vitamin B production, which may lead to improve early infant growth and birth weight [56].

Three case–control studies assessing gut microbiome in gravidas with FGR broadly depicted similar compositional and gene functions profiles in FGR compared with controls matched by age and non-FGR clinical characteristics [46,47,48]. These profiles also broadly aligned with the dysbiosis and functional potential alteration in human neonates and newborn/developing animal models with FGR [41,42,43,44]. Similar to the newborn animal studies, it was inconclusive whether any differences existed in the overall composition of the gut microbiomes between FGR gravidas and controls [46,47,48]. In the early rodent model reviewed, statistically indifferent quantities of common gut genera were reported between pregnant dams with diet induced FGR and controls, although the former group had lower total bacterial load [42]. Whether such inconsistency was due to different DNA extraction and sequencing methods and bioinformatics tools requires further analysis [57]. Despite this uncertainty, the taxa that were found to be more prevalent in FGR gravidas or associated with maternal and neonate FGR clinical features tended to be those capable of influencing host macronutrient or energy metabolism. The involvement of the gut microbiota in host macronutrient and energy metabolism and inflammation in FGR gravidas was supported with an analysis of predicted [47,48] and actual [46] microbial gene function pathways. For example, amino acid metabolism, lipid metabolism, glycan biosynthesis, and energy metabolism, that potentially contribute to the Kyoto Encyclopedia of Genes and Genomes (KEGG) pathway [48], carbohydrate metabolism to host energy metabolism [47], and the amoebiasis pathways [46], were found to be augmented in FGR gut microbiomes. Suppressed gut microbial nitrogen metabolism was demonstrated in FGR gravidas in comparison with controls. This abnormality may contribute to either reduced nitrite-mediated vasodilation in placenta or amino acid metabolism, impeding the host’s nutritional status and fetal development. This alignment between gut microbiome compositional and functional capacity alteration was broadly in line with observations in both rodent [42] and swine [44] models that examined newborn animals.

A more complicated cascade of events leading to FGR was proposed by Hu et al. [49] and Stupak et al. [50] who studied placental microbiomes in FGR gravidas. Hu et al. [49] outlined that an elevation of pathogenic bacteria such as *Neisseriaceae* in the maternal oral microbiome, rather than the gut microbiome, could create hypoxia by binding to iron-bound host proteins. This low-oxygen environment impedes the colonization of potentially beneficial *Bifidobacteraceae* and alters energy metabolism in facultative anaerobes such as *Lactobacillaceae* to produce lactate and ethanol. These metabolites can impact on host mitochondrial activity and ultimately uteroplacental insufficiency, and hence FGR. These alterations, together with proliferation of strict anaerobes lead to placental microbiome dysbiosis that may be prone to the colonization of opportunistic pathogens, which further propels this vicious cycle. In a study by Stupak et al. [50], microbiological screening of the placenta showed significantly higher relative abundances of pathogenic bacteria (e.g., *E. coli*, *Listeria costaricensi*, *Clostridiales bacterium*) in the FGR group when compared with the controls. Thus, the exact source of maternal inflammation that triggers FGR development awaits further investigation. This is particularly interesting considering the oral microbiome may also serve as a microbial reservoir for the gut microbiota [58]. Irrespective of the microbial source, it seems plausible to propose the existence of a placenta–gastrointestinal tract microbiome (oral, intestinal, or colonic) axis, where dysbiosis in the latter compartment seems to impact on the reproductive microbiome mediated by components and/or metabolites of certain microbes. The reproductive microbiome dysbiosis is accompanied by pathological changes in the placenta that eventually leads to manifestations of FGR. Human studies that have assessed the alterations of gut microbiota in FGR cases are summarized in Table 2.

## 4. Inflammation in FGR

Inflammation is considered as a potential mechanism in FGR but has not yet been examined in relation to the gut microbiota profiles in humans. At present, both animal and human studies have assessed the pro-and anti-inflammatory cytokine levels in FGR. The limited evidence available in animal models suggests that inflammation is associated with the relative abundances of certain gut microbiota taxa.

### 4.1. Animal Models

A recent study reported that the gut microbiota colonization in FGR piglets during the first 12 h after birth induces a plasma cytokine imbalance. FGR piglets with a high relative abundance of *Escherichia-Shigella* and a low abundance of *Clostridium_sensu_stricto_1* are at higher risk of developing inflammation [59]. IUGR piglets are more susceptible to infections than normal piglets, which could in turn lead to impaired intestinal mucosal immunity from birth, resulting in damaged intestinal morphology and decreased levels of Th1 cytokines in the ileum/Jejunum. A partially defective innate immune system and the presence of lower total T-cells may explain the decreased levels of cytokines [60]. Serum levels of LPS-induced IL-1β were found to decrease in the peripheral blood mononuclear cells (PBMCs) of IUGR piglets. This decrease is not beneficial and probably occurs due to a diminished immune response [61].

In vivo experiment showed that the stimulation of toll-receptor 9 (TLR9) by LPS in FGR pregnant rats was associated with the relative abundances of Prevotellaceae, *Streptococcus*, *Prevotella*, and *Bacteroides*. This was observed through the activation of multiple signaling pathways, such as tumor necrosis factor receptor associated factor 3 (TRAF3), TANK-binding kinase 1 (TBK1), and interferon regulatory factor 3 (IRF3) [62]. Maternal food restriction during pregnancy causes IUGR and reduces the immunotolerant milieu in mouse placenta, which may affect immune tolerance and the immunologic pathways regulating ER stress and autophagy [63]. Prenatal immobility stress at different periods of pregnancy can cause IUGR in rodents, which results in increased levels of pro-inflammatory cytokines and decreased levels of antioxidant enzymes [64]. The administration of FK565, an innate immune receptor of Nod1, to pregnant C57BL/6 mice resulted in increased IUGR risk, which, in turn, can activate genes associated with inflammation and immune responses in fetal vascular tissues [65]. LPS administration to FGR rats resulted in increased placental circulating pro-inflammatory cytokine levels and hypoxia-inducible factor 1-alpha (HIF-1α) accumulation [66]. Maternal inflammation at mid-gestation may contribute to reduced skeletal muscle growth and fetal myoblast functions in IUGR rodent pups [67]. An experimental study has shown evidence that myoblasts isolated from fetal IUGR sheep display aberrant proliferation and systemic inflammation via stimulation of the inflammatory signaling pathways [68]. Findings from animal models examining the inflammatory markers in IUGR are summarized in Table 3.

### 4.2. Human Studies

The exact mechanisms mediating the relationships between pro-inflammatory cytokines and gut microbiota in FGR gravidas have not yet been fully elucidated [69]. A systematic review of human studies showed that preterm birth is characterized by increased markers of inflammation, and occurs as a result of the influence of several risk factors such as placental abruption, intrauterine infection, lower genital tract infection, cervical shortening, anemia, and smoking [70]. Genetic factors also play a significant role in increasing susceptibility to inflammation in preterm infants, in which heritable changes in gene expressions result from TLR5 overexpression, leading to increases pro-inflammatory cytokines/chemokines [70]. Maternal OS and inflammation markers in pregnancy (e.g., 8-isoprostane and 8-hydroxydeoxyguanosine, IL-6, IL-10, IL-1β, and TNF-α) have been found to be associated with decreased fetal growth [71]. The reduced numbers of plasmacytoid peripheral blood dendritic cells (DCs) in IUGR pregnant women may lead to increase pro-inflammatory cytokines/chemokines production [72].

It was postulated, based on findings from a few studies, that aberrant inflammation in gravidas and/or newborns could lead to IUGR (Table 4). An imbalance between pro and anti-inflammatory cytokines was observed in IUGR pregnant women with placental insufficiency. The results indicate an increase in the ratio of pro-inflammatory to anti-inflammatory cytokines in IUGR women with placental insufficiency compared to normal pregnant women [73,74,75]. A study reported that the serum levels of pro-inflammatory cytokines were higher in IUGR gravidas compared with healthy counterparts, but the difference was not statistically significant [76]. Another study reported that idiopathic IUGR mothers had higher inflammation markers than appropriate-for-gestational-age (AGA) infants. IUGR contributes to changes in uteroplacental hemodynamics by increasing the serum levels of pro-inflammatory cytokines, including adrenomedullin [77]. Serum levels of pro-inflammatory cytokines were found to be higher in IUGR infants than AGA infants. Increasing IL-6 and IL-18 levels in IUGR infants indicate the existence of inflammation [78]. IUGR infants had higher pro-inflammatory cytokine levels, including IL-6 and TNFα compared with AGA and SGA infants [79].

Since FGR is a process with increased inflammatory markers, fluctuations in some markers related to inflammation in human and animal studies may be indicated by inflammation-related mechanisms. Further studies to clarify the mechanisms underlying inflammation-induced FGR, and the relationships among inflammation markers and their expressions, are needed.

## 5. Effects of Probiotics in FGR

Possibly reflecting the limited number of observational studies in animal models and humans, the efficacy of probiotic therapies in ameliorating FGR in newborns and mothers seems to have been reported in only a few studies [54,80,81,82,83,84]. The level of heterogeneity in study design also appears to be similar to the observational studies reviewed. The targets of intervention seem to more or less follow findings from the observational studies. These include stimulating intestinal development in the FGR offspring to increase digestion and absorption capacity and immune function [54,80], and regulating liver function [81]. Another approach aimed to restore fetal–placenta development in the pregnant mother to ameliorate alterations in the growing environment for the fetus [81]. Both approaches aimed to promote fetal growth and development.

### 5.1. Animal Models

Two animal models, one swine [54] and one rodent [80], were found to investigate the efficacy of probiotics in newborn growth and intestinal development. One murine model focused on maternal responses in terms of body composition, major metabolic and immune organs, caecum metabolites, and hormone levels in blood, in addition to fetal liver function [81]. The *Bacillus subtilis* PB6 strain used in a swine model [54] and the two breast milk-derived *Lactobacillus* strains *Limosilactobacillus oris* ML-329 (ML-329) and *Lacticaseibacillus paracasei* ML-446 (ML-446) administered to a rodent model [80] demonstrated their viability and increased abundance in the gut lumen. Both studies observed significant morphological and physiological improvement in the intestine in FGR neonates. The intestinal length and weight of FGR rats supplemented with ML-329 and ML-446 were superior to their corresponding blank controls [80]. Increased villous height [54,80] and reduced villi crypt ratio [54] were also reported. The latter indicates a better capacity for digestion and absorption. The accompanying physiological improvements, such as elevated maltase and sucrose activity levels in FGR piglets irrespective of body weight, added to the efficacy of the *B. subtilis* PB6 strain. Improved intestinal development was also accompanied by an augmented feeding efficiency, as represented by a significant reduction in feed conversion ratio, but not on body weight and organ-related growth parameters. The effects on improving intestinal barrier function were highlighted by elevated expression of genes for proteins crucial to intestinal tight junctions such as zonula occludens-1 in both FGR and control newborn piglets. The rodent model revealed that the aforementioned breast milk-derived *Lactobacillus* strains were able to stimulate the expression of genes involved in the Wnt signaling pathway and suppress those expressed in the Notch signalling pathway, and thus promoting the proliferation and differentiation of intestinal epithelial cells [80]. Among them, the mucin-producing goblet cells and the immunoregulatory Paneth cells are critical in maintaining intestinal barrier integrity and innate immunity [85,86]. These two animal models demonstrated the possibility that impaired intestinal development could be at least partially rescued by administrating probiotic strains known for their efficacy in stimulating gut tissue and immune function development. The possibility to improve fetal growth by promoting placenta development was underlined by findings from a murine model which was supplemented with the strain *Bifidobacterium breve* UCC2003/pCheMC following a pattern that took advantage of the natural elevation of *Bifidobacterium* sp., during pregnancy [81]. Maternal body composition and the subsequent organ responses to supplementation were modified, accompanied by increases in caecum and/or placenta carnitine and acetate. The former could affect energy metabolism and the latter is known to mediate host glucose metabolism and intestinal epithelial cell immune function. These changes were postulated to enable resource allocation to fetus. The reduction in the thickness of the interhaemal membrane barrier of the placenta could also contribute to fetal growth by diminishing the barrier to exchange resources between the placenta and the fetus. Further facilitating the maternal supply of resources to the fetus, the expression of genes involved in glucose and fatty acid transporters in placenta was augmented. These preliminary data seem to support the supplementation of probiotic strains during pregnancy at risk of FGR. Considering the lack of statistically significant improvement in newborn body weight reported by those animal models reviewed, studies supplementing multiple strains with unique verified effects on maternal energy metabolism and inflammatory status, placenta development, and fetal/infant intestinal development may help determine the clinical potential of probiotics therapy in FGR management [81]. A consensus in study design regarding the factors influencing the consistency of the results between observational studies summarized in Section 3 is warranted. Appropriately labelled probiotic strains or their metabolites may help confirm the cause and effects and/or trace the pathways involved in mediating the effects in these studies [81]. Findings from animal models on probiotic supplementation in FGR treatment are listed in Table 5.

### 5.2. Human Studies

The consumption of probiotic foods may provide beneficial effects in reducing prematurity complications during pregnancy. However, whether probiotic supplementation can reduce FGR in humans remains an ongoing debate. Evidence from a prospective cohort study over a 9-year period suggests that probiotic milk intake during late pregnancy was associated with a decreased risk of PE. Probiotic intake during early pregnancy was associated with a reduced risk of preterm delivery [87]. Another cohort study has demonstrated the potential of milk-based probiotic products to reduce the risk of PE in primiparous pregnant women over a 6-year period [88].

Clinical studies in humans seem to demonstrate even less optimistic results in its efficacy [82] and risk of adverse effects [83,84] A double-blinded RCT in FGR and normal weight infants born before 33 weeks of gestational age reported limited clinical benefits of *Bifidobacterium breve* M-16V supplementation until the age of 37 weeks. The only improvement reported was that those with FGR who were supplemented were able to reach full feeds a couple of days earlier than the FGR placebos. This was despite the significant increase in fecal counts of *B. breve* among the supplemented group in comparison with those not supplemented irrespective of their FGR status. This outcome was among a number of parameters tested including a comparison of total fecal *B. breve* counts 3 weeks post supplementation between FGR and normal weight controls, and a number of different growth and clinical parameters. It is unclear whether increasing the sample size or altering the probiotic strains administered would lead to better outcomes [82]. In addition to limited clinical benefits, safety concerns regarding probiotic supplementation in FGR infants were cautioned in two case reports [83,84]. In one case, *Lactobacillus rhamnosus* GG (LGG) ATCC 53,103 (3 × 10^9^ cfu, once a day) [83] was administered to prevent antibiotics-associated gut complications, in the other an unspecified *L. rhamnosus* GG strain (0.5 × 10^9^ CFU/day) [84] was administered to prevent NEC. Sepsis with bacteria identified to match the supplemented strain was diagnosed in both cases [83,84]. Bacterial translocation across the intestinal barrier in severely ill FGR infants was suspected in both cases. While neither case identified the exact route of infection, the more recent case highlighted the possibility of contamination of the central venous catheter which contained glucose that potentially contributed to bacterial growth [83]. The establishment of safety protocols for prescription and administration of probiotic products in those suspected of impaired immune function including FGR infants, particularly those with glucose infusion, are warranted [83,84]. Table 6 summarizes findings from human studies that have evaluated the effectiveness of probiotic supplementation in FGR treatment.

Current evidence from experiments in animal models and human trials, albeit scarce, indicates the plausibility and potential of probiotic strains supplementation in either the newborn or the mother to ameliorate impaired growth in FGR. Supplementation targeting infant gut microbiota appeared to mediate an improvement in intestinal development and immune function and possibly feeding efficiency. Therapies provided during pregnancy showed promising efficacy in partially restoring fetus–placenta exchange and fetal growth environment, both critical to support fetal development. The minimal efficacy of reaching full enteral feed a couple of days earlier in the one human trial found implies the need for more sophisticated clinical trials addressing the multiple limitations in study design identified in both animal and human studies. The two case reports of probiotic-associated sepsis highlighted clinical caution and evidence-based guidelines required when adopting probiotic therapies.

## 6. Conclusions

This review articulated the mediating role of gut microbiota and inflammation in the manifestations of FGR. Despite the broadly consistent patterns observed in human and animal studies, many inconsistent findings were present, even within the animal or human studies, particularly at lower taxonomic and gene function category levels. The main factors that could have contributed to such inconsistencies include the selection of different animal models and study designs, the adoption of different definitions of FGR, sampling times, microbiome quantification approaches and sequencing methods and depths, microbiome sampling approaches, and anatomical sources of microbiomes.

The current review corroborates prior evidence that maternal inflammation is associated with the development of FGR, focusing on both human studies and animal models. The relationship between gut microbial abundance and FGR pathogenesis has been identified in two animal models. High relative abundances of *Escherichia-Shigella*, Prevotellaceae, *Streptococcus*, *Prevotella*, and *Bacteroides* in FGR piglets/rats may induce inflammation. Further studies are needed to highlight the pro-inflammatory and/or anti-inflammatory effects of gut microbial taxa in FGR gravidas and animal models.

Evidence is emerging that perturbations in maternal microbiota, whether originating from gut or oral cavity, could lead to adverse fetal growing environments and impaired resources supply induced by inflammation and/or hypoxia directly or indirectly impacting on the placenta. The existence of a gut–placenta–fetus axis seems plausible. Probiotic strains with verified efficacy in promoting development of the infant intestine, gut associated immune function, and energy harvest capabilities could potentially contribute to the alleviation of FGR manifestations in the infants. Probiotics supplemented during pregnancy may help improve the fetal growth environment and maternal–fetal exchanges essential to restoring fetal organ and immune function development. More sophisticated studies testing multiple strains targeting both the newborn and maternal microbiota may help determine the type and extent of clinical efficacy and effectiveness, the target of administration (i.e., infant and/or mother), and the safety of probiotic therapies in FGR treatment. More studies are also needed to investigate whether probiotics modulate the gut microbiota composition and inflammation for FGR treatment.

## Figures and Tables

**Table 1:** Summary of animal models that have evaluated the alterations of gut microbiota in FGR cases.

Experimental Model	Sampling	Bacterial Taxonomic Expressions	Ref.
Rats	Four pregnant Sprague Dawley rats FGR (rats fed a diet containing 7% protein until birth) and controls (rats fed a diet containing 21% protein) Fecal samples were collected using polymerase chain reaction amplification and sequencing	FGR compared with controls *Enterococcus*, *Blautia*, Enterobacteriaceae ↑ *Clostridium*, *Eubacterium Akkermansia*, *Allobacutum* ↓	[41]
Rats	Virgin Sprague Dawley rats (IUGR and controls/normal birth weight Cecocolonic contents were analyzed using real-time quantitative polymerase chain reaction (qPCR) at different ages (days 5, 12, 16, 22, 40, and 100)	IUGR compared with controls Day 5 = *Bacteroides* sp., clostridial cluster IV, *C. leptum cluster*, *F. prausnitzii*, *R. intestinalis* ↑ Day 12 = *Bifidobacterium* sp., clostridial clusters IV and XIVa ↓ Day 16 = *Lactobacillus* sp., clostridial cluster XIVa ↑ *E coli* ↓ Day 40 = *Bifidobacterium* sp. ↓ *Bacteroides* sp. ↑ Day 100 = *R. intestinalis* ↑	[42]
Piglets	48 newborn large white and landrace piglets = (24 IUGR and 24 controls/normal birth weight) Microbial community structure was analyzed at 7, 21 and 28 days of age Jejunum and ileum content samples were analyzed using a thermocycler PCR system	IUGR compared with controls Days 7, 21 and 28 = Bacteroidetes, *Bacteroides* ↓ Day 21 = *Oscillibacter*, Firmicutes ↓ Proteobacteria, *Pasteurell* ↑ Day 28 = *Escherichia-Shigella* ↑	[43]
Piglets	36 large white and landrace piglets = (18 IUGR and 18 controls/normal birth weight) Intestinal microbiota composition was analyzed when pigs reached 25, 50, and 100 kg of body weight PCR amplification was used to analyze the duodenum-jejunum contents	IUGR compared with controls 25 kg BW group = Bacteroidetes ↑ Proteobacteria, *Lactobacillus* ↓ 50 kg BW group = Firmicutes ↑ Proteobacteria, Thermi, Cyanobacteria, Bacteroidetes ↓ 100 kg BW groups = Firmicutes ↑ *Fusobacteria*, Proteobacteria, Thermi ↓	[44]

(↓) decrease, (↑) increase.

**Table 2:** Summary of human studies that have evaluated the alterations of gut microbiota in FGR cases.

Study Design	Sample Characteristics	Bacterial Taxonomic Expressions	Ref.
Prospective cohort study	2~3 years of follow-up 150 twin neonates classified into four groups Fecal samples were used to characterize the gut microbiota using 16S ribosomal RNA (rRNA) sequencing metabonomics and metagenome sequencing Groups: monochorionic-diamniotic (MCDA) twins with birth weight discordance/FGR (30 cases), MCDA twins with birth weight concordance (43 cases), dichorionic-diamniotic (DCDA) twins with birth weight discordance (26 cases) and DCDA with birth weight concordance (51 cases)	MCDA-C compared with other groups = *Actinobacillus* ↑ MCDA-C group compared with MCDA-D = *E. faecium* ↑ MCDA-D and MCDA-C groups compared with DCDA-C and DCDA-D groups = *Coprococcus*, *Robinsoniella*, *Oscillospira* ↑ *Acinetobacter*, *Enterococcus*, *Actinobacillus* ↓	[45]
Case–control study	16 gravidas = eight FGR and eight controls/normal Metagenomic sequencing and bioinformatic analysis on the fecal samples of gravidas were used	FGR compared with controls = *Fusobacteria*, *Aerophobetes*, *Ornatilinea*, *Sphingomonas*, *Plesiomonas* ↑ *Roseburia*, Prevotellaceae, *Dysgonomonas*, *Anaerovibrio*, *Mobilisporobacter*, *Symbiothrix*, *Arthromitus*, *Micrococcales*, *Thermohydrogenium*, *Cellulosimicrobium*, Propionibacteriaceae, *Aquabacterium*, *Roseomonas* ↓	[46]
Case–control study	32 gravidas = 14 FGR and 18 controls/normal Fecal samples were obtained from maternal rectum using 16S rDNA amplicon sequencing	FGR compared with controls = Firmicutes, *Bacteroides*, *Faecalibacterium* and *Lachnospira* ↑	[47]
Case–control study	70 gravidas = 35 FGR and 35 controls/normal Fecal samples were using 16S rRNA sequences and metabolomes	FGR compared with controls = *Lactobacillus*, *Catenibacterium* ↑ Ruminococcaceae, *Bacteroides uniformis*, *Mollicutes*, *Alistipes onderdonkii* ↓	[48]
Case–control study	40 gravidas = 20 IUGR and 20 controls/normal 16S rRNA Sequencing was used to analyze the reproductive microbiome	IUGR compared with controls = *Neisseriaceae*, *Desulfovibrio* ↑ Firmicutes/Bacteroidetes ratio, *Bifidobacterium*, *Lactobacillus* ↓	[49]
Case–control study	36 gravidas = 18 FGR and 18 controls/with physiological pregnancy and eutrophic fetus Liquid chromatography–mass spectrometry (LC-ESI-MS/MS) was used to analyze the placental microbiome	FGR compared with controls = *Actinopolyspora erythraea*, *Pararhizbium haloflavum*, *Clostridiales bacterium*, *Paenisporosarcina* sp., *Acidobacteria bacterium*, *Escherichia coli*, *Mucilagini bacteria* sp. *Shinorhizobium arboris* ↑	[50]

(↓) decrease, (↑) increase.

**Table 3:** Summary of animal models that examined inflammation in IUGR.

Experimental Model	Sampling	Inflammatory Markers	Ref.
Piglets	12 sows (6 IUGR and 6 controls/normal birth weight)	IUGR compared with controls = Interleukins (IL)-4, IL-1β, interferon (IFN)-γ, tumor necrosis factor (TNF-α) ↑ IL-2, IL-10, Immunoglobulins IgG and IgA ↓	[59]
Piglets	12 newborn Duroc × (Landrace × Yorkshire) piglets (six IUGR and six controls/normal birth weight)	IUGR compared with controls = IL-2, IL-1β, IFN-γ, TNF-α ↓	[60]
Piglets	40 Danish Landrace × Danish Yorkshire piglets (20 IUGR and 20 controls/normal birth weight)	IUGR compared with controls = IL-1β ↓	[61]
Rats	10 female Sprague Dawley rats were assigned to two groups: FGR group (n = five) and healthy group (n = five)	FGR compared with controls = IL-1β, TNF-α, TRAF3, TBK1, IRF3 ↑ IL-10 ↓	[62]
Rats	Male and female C57/BL6 pregnant mice were assigned to three groups: mild restriction group-restricted by 25% of their daily intake, moderate restriction group-restricted by 50% of their daily intake, and ad libitum access to the standard rodent diet	Groups 1 and 2 compared with group 3 = IL-10 ↓	[63]
Rats	50 adult pregnant virgin female Wistar albino rats were assigned to five stress groups: Groups I and II (Day 1–10 stress group), Group III and Group IV (10–19th-day) and Group V (1–19th-day stress group)	Groups III, IV and V compared groups I and II = IL-6, TNF-α ↑ IL-10 ↓	[64]
Rats	Pregnant C57BL/6 adult female mice	IL-6, TNF-α, CC chemokine ligand 2 (CCL2) ↑	[65]
Rats	13 pregnant virgin female Wistar rats were assigned to three groups: LPS (n = seven dams per time-point), Saline (n = three dams per timepoint), and LPS + Goniothalamin (GTN) (n = three dams per timepoint)	LPS group compared to other groups = IL-6, IL-10, TNF-α, HIF-1α ↑	[66]
Rats	19 IUGR pregnant Sprague-Dawley rats were assigned to be injected with saline (n = nine, controls) or 0.1 µg/kg BW of LPS endotoxin from *E. coli* (n = 19, cases)	Cases compared to controls = TNF-α, tumor necrosis factor receptor 1 (TNFR1), IL6R (IL-6 receptor) ↑	[67]
Sheep	Experiment 1: nine IUGR ewes were exposed to ambient temperatures (40 ± 1 °C, 35 ± 5% relative humidity); seven normal ewes were exposed to temperatures at 25 ± 1 °C and 35 ± 5% relative humidity. Experiment 2: six IUGR ewes were injected with LPS, endotoxin and *E. coli*; seven normal ewes were injected with saline carrier.	Experiment 1: IUGR compared with controls = IL-6, TNF-α, IκB-Kinase ↑ Experiment 2: IUGR compared with controls = TNF-α, IκB-Kinase, β-catenin, nuclear factor kappa B (NF-κB), TNFR1 ↑	[68]

(↓) decrease, (↑) increase.

**Table 4:** Summary of human studies that examined inflammation in IUGR.

Study Design	Sample Characteristics	Inflammatory Markers	Ref.
Case–control study	58 gravidas = 36 IUGR and 22 controls/normal	IUGR compared with controls = IL-4, IL-6, IL-12, TNFα ↑ IL-10 ↓	[73]
Case–control study	58 gravidas = 36 IUGR and 22 controls/normal	IUGR compared with controls = IL-8, IL-12, IFNγ, TNFα ↑ IL-10, IL-13 ↓	[74]
Case–control study	100 gravidas = 50 IUGR and 50 controls/normal	IUGR compared with controls = IL-6, TNF-α, high-sensitivity C-reactive protein (hsCRP), erythrocyte sedimentation rate (ESR) ↑ IL-10 ↓	[75]
Case–control study	80 gravidas = 20 PE, 24 IUGR and 36 controls/normal	IUGR and PE compared with controls = IL-6, hs-CRP ↑	[76]
Case–control study	75 participants = 50 idiopathic IUGR and 25 AGA infants	Idiopathic IUGR compared with AGA = IL-6, TNF-α, adrenomedullin ↑	[77]
Case–control study	100 infants = 50 IUGR infants and 50 AGA infants	IUGR compared with AGA = IL-6 and IL-18 ↑	[78]
Retrospective study	140 participants = 37 IUGR mother-child couples/fetuses, 70 AGA and 33 SGA infants	IUGR compared with AGA and SGA = IL-6, TNFα, leptin ↑ Adiponectin ↓	[79]

(↓) decrease, (↑) increase.

**Table 5:** Summary of animal models that investigated the effects of probiotic supplementation in FGR treatment.

Experimental Model	Probiotic Strains/Prebiotics	Dosage	Duration	Effects	Ref.
Piglets	*Bacillus subtilis* PB6	60 g per 100 kg FORM powder, containing 2 × 10^9^ cfu/kg.	21 days	Increased protein and mRNA abundances of claudin-1 and zonula occludens-1 in the intestine Decreased plasma levels of IL-1β, immunoglobulin A (IgA), percentage/count of blood lymphocytes, maltase activity, villous height, Toll-interacting protein and mRNA abundance of TLR-9 in the intestine	[54]
Rats	*Limosilactobacillus oris* ML-329, *Lacticaseibacillus paracasei* ML-446, *Lacticaseibacillus rhamnosus* GG	100 μL of PBS (Solarbio) or strains in PBS (1 × 10^7^ CFU) (Solarbio)	1–10 days	ML-329 and ML-446 compared with *Lacticaseibacillus rhamnosus* GG = Increased the number of goblet and Paneth cells in the intestine, the mean density of lysozyme, the expression of wnt gene and the abundance of β-catenin protein Decreased the expression of Notch gene	[80]
Rats	*Bifidobacterium breve* UCC2003	100 μL of reconstituted lyophilised *B. breve* (containing 10^10^ CFU/mL)	10–14 days	Altered metabolites/nutrient milieu, placental structure, maternal body adaptations and the expression of signaling pathways implicated in cell proliferation	[81]

**Table 6:** Summary of human studies that investigated the effects of probiotic supplementation in FGR treatment.

Study Design	Probiotic Strains	Sample Characteristics	Effects	Ref.
RCT	*Bifidobacterium breve* M-16V	42 FGR infants supplemented with probiotic (22 cases) or dextrin (Placebo, 20 cases) 111 non-SGA preterm infants supplemented with probiotic (55 cases) or dextrin (Placebo, 56 cases)	Reached full feeds in FGR infants	[82]
Case report	*Lactobacillus rhamnosus* GG (ATCC 53103	Female preterm infant treated with probiotic (3 × 10^9^ cfu, once a day)	Developed sepsis after the treatment	[83]
Case report	*Lactobacillus rhamnosus* GG (ATCC 53103	6-day-old IUGR newborn treated with probiotic (0.5 × 10^9^ CFU/day)	Increased inflammation parameters (high levels of serum C-reactive protein (CRP), procalcitonin and immature neutrophils/neutrophils). Decreased white blood cell count/platelet count	[84]

## Data Availability

Not applicable.
